# Heterogeneous estimations of non-pharmaceutical mitigation behavior during the COVID-19 pandemic

**DOI:** 10.1038/s41597-026-07348-3

**Published:** 2026-05-02

**Authors:** David J. Butts, Nidhi Parikh, Sara Y. Del Valle

**Affiliations:** Los Alamos National Laboratory, Analytics, Intelligence and Technology Division, Los Alamos, 87545 USA

**Keywords:** Diseases, Health care

## Abstract

The COVID-19 pandemic highlighted the importance of human behavior in mitigating the spread of disease. Nonetheless, human behavior is often overlooked in models of disease spread, particularly by underutilizing real-world data. We address this by estimating probabilities that individuals engage in behaviors that influence SARS-CoV-2 transmission risk during the COVID-19 pandemic, between September 2020 and June 2022. These behaviors include wearing a mask, using public transportation, spending time with others, avoiding contact with others, and going to work. Our estimates account for the age and sex of individuals and are generated for every county in the United States. We utilized multiple open-source datasets and United States Census data to produce these estimates. Multiple datasets were used for validation, showing our estimates demonstrated comparable accuracy and robustness. Our estimates aid in understanding human behavior dynamics during the COVID-19 pandemic and could be used to inform monthly or longer-term behavior in simulations of COVID-19. Moreover, the methods presented can be applied to other behaviors and features for future simulations of infectious disease.

## Background & Summary

For millennia, infectious diseases have posed significant challenges to humanity, yet scientific advancements have deepened our understanding of the factors driving their spread, including host-pathogen dynamics and human-to-human interactions. For example, epidemic models–such as Susceptible-Infectious-Recovered (SIR) model, a system of ordinary differential equations^1,2^, have long served as foundational tools for studying disease transmission and control^3–6^. Advances in computational power have further transformed the field, enabling the development of more intricate models, such as agent-based models (ABMs)^7–9^, which allow for the simulation of heterogeneous populations and interactions.

Despite these advances, many models have been limited in their incorporation of human behavior. While the inclusion of behavior is critical for understanding epidemic dynamics, its integration into modeling frameworks has been relatively slow^10–12^. Most behavioral models tend to be theoretical, with limited validation against real-world data^13^ and many fail to use observational data for parameterizerization^14^. This lack of behavioral considerations can produce inaccurate projections of the progression of a disease, undermining the credibility of modeling efforts^12^ and in some cases, eroding public trust. Nevertheless, disease models that incorporate behavior have offered valuable insights into complex disease dynamics. These include phenomena such as subexponetial growth dynamics^10,15^, the effect of social interactions on epidemic sizes^16^, and the emergence of multiple infection peaks^17,18^. These models demonstrate the importance of accounting for behavioral feedback mechanisms to capture the true complexity.

The COVID-19 pandemic, however, underscored the critical influence of human behavior on the progression of epidemics when choices about work closures and wearing masks were required and generated an unprecedented amount of data to help quantify and better understand its impact. Throughout the pandemic, many researchers employed surveys to track aspects of human behavior such as mobility^19^, and various mitigation behaviors such as mask wearing, hand washing, and social distancing^20–27^. These surveys vary spatially from county- to national-levels and temporally from days to years, often capturing demographic factors such as race, age, and sex. The extensive coverage across regions and time allows for behavior to be studied at multiple levels of resolution.

While these surveys provide invaluable insight into human behavior during the pandemic, they are difficult to use directly in epidemiological models due to missing data, response biases, and sample biases. Different types of biases can lead to erroneous results, and has been observed in large COVID-related surveys^28^. We correct for missing data using the machine-learning-informed imputation method, MissForest^29^. We utilize data from multiple sources to complement missing data and reduce bias from any single source. In particular, we use open-source data from The Delphi Group at Carnegie Mellon University U.S. COVID-19 Trends and Impact Survey, in partnership with Facebook (Delphi US CTIS)^20^, COVID States project^21^, and United States Census^30–32^ to generate estimates of the percent of individuals that wore a mask, went to work, used public transit, spent time with others, and avoided contact with others during the COVID-19 pandemic between September 2020 and June 2022 at the state and county levels by age and sex. We employ the iterative proportional fitting (IPF) algorithm^33–35^, specifically, a two-step IPF process similar to the one described by Beckman *et al*.^36^ to connect data from multiple spatial scales.

We anticipate that our estimates of mitigation behavior uptake during the COVID-19 pandemic can inform human behavior in models that further explore the pandemic. Providing these estimates directly will aid researchers by saving time and effort in producing estimates for their own studies. Moreover, we present our methods in detail so that researchers can apply them to behavior in other settings.

## Methods

**Fig. 1:**
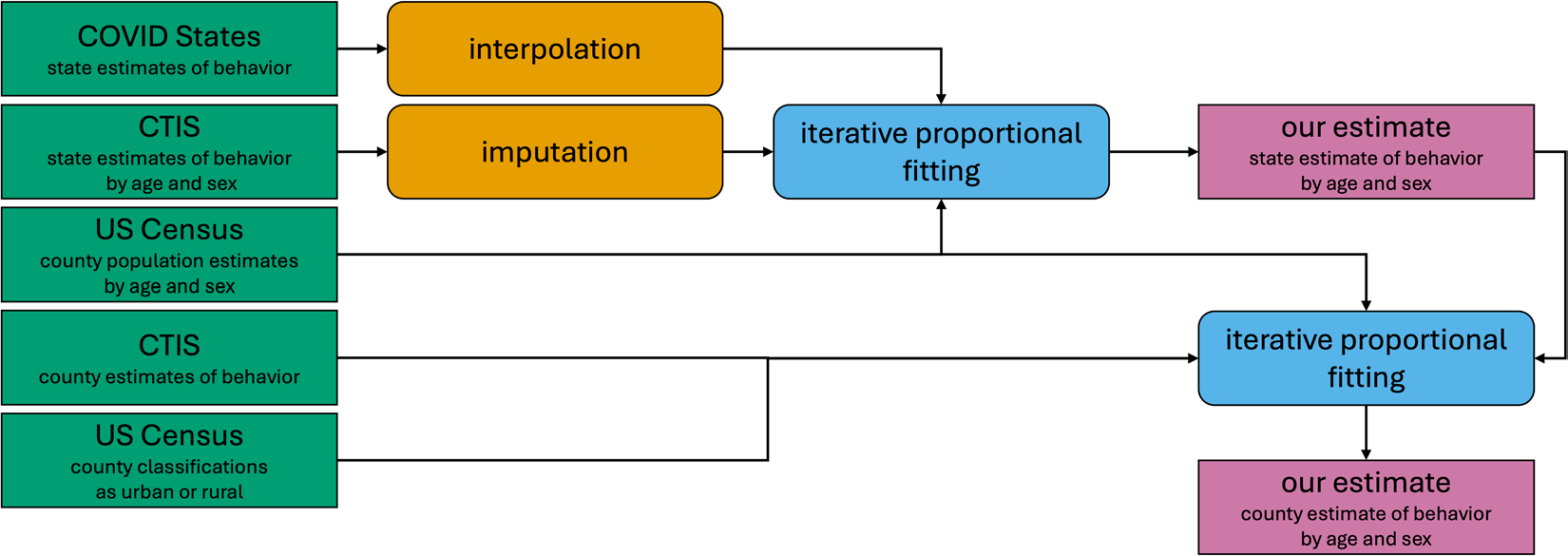
Flowchart describing estimate generation for mitigation behavior uptake during the COVID-19 pandemic. Raw inputs (**left-most green boxes**) that contain state-level data are cleaned using interpolation and imputation (**orange rounded boxes**). These cleaned data and other inputs are used as input to a two step IPF process (**blue rounded boxes**) to produce state- and county-level estimates (**purple** **boxes**) of mitigation behavior uptake.

We outline our processes for generating estimates of behavior uptake in Fig. 1. Raw data inputs are represented with green boxes, on the left of the diagram. The inputs that require data cleaning, state-level data from COVID States and Delphi US CTIS, are input to data cleaning methods shown with orange rounded boxes. The resulting cleaned data and the remaining input data are used in the two IPF steps shown with blue rounded boxes. These IPF steps produce state- and county-level estimates shown with purple boxes. The first IPF step generates state-level estimates that the second step uses to produce the county-level estimates. We provide details for each of these steps in the following subsections.

### Datasets

We gathered open-source data from the Delphi US CTIS^20^, COVID States project (https://lazerlab.shinyapps.io/Behaviors_During_COVID)^21^, and United State Census^30–32^ to inform our predictions. Additional data from Sanchez *et al*.^22^, Taube *et al*.^37^, and YouGov (https://yougov.com/en-us/articles/29430-personal-measures-taken-avoid-covid-19)^23^ were used to validate our estimates. For convenience, we list all of the datasets we used, and their use cases in Table 1.

**Table 1:** Datasets used in this study to estimate behavior uptake by county, age group, and sex.

Name	Data use	Source
monthly_county_theme_behavior_and_mental_health_overall	county-level estimates of mitigation behaviors without demographic information	Delphi US CTIS^20^
monthly_state_all_indicators_agefull_sex	state-level estimates of mitigation behaviors by age and sex	Delphi US CTIS^20^
behaviors_during_COVID	state-level estimates of mitigation behavior	COVID States^21^
cc-est2023-agesex-all	age and sex distribution by county 2022–2023	US Census^31^
cc-est2021-agesex-all	age and sex distribution by county 2020–2021	US Census^30^
2020_UA_COUNTY	classification of US counties as urban or rural	US Census^32^
Sanchez *et al*.	national-level mask wearing by sex	Sanchez *et al*.^22^
personal measures taken to avoid COVID-19	national-level mask wearing, not going to work, avoiding other in public	YouGov^23^
Taube *et al*.	national- and state-level mask wearing	Taube *et al*.^37^

The first six rows describe data that informed our estimates of behavior, while the final three rows describe data used for validation.

The Delphi US CTIS provides data at two spatial aggregations: state and county. The state-level aggregation estimates the percent of individuals that belong to a demographic profile who engage in various mitigation behaviors, while the county-level data does not distinguish by demographics. For this study, we focus on two demographic factors: sex and age. Though the Delphi US CTIS estimates a wide range of behaviors, we focus on its estimates of individuals who wear a mask outside their homes, work outside their home, avoid contact with others, use public transportation, and spend time with people outside their household. Behavioral data is available at weekly or monthly time aggregates; due to higher variability in data quality in weekly aggregates, we chose to use the more stable monthly data.

Unlike the state-level Delphi US CTIS data, the COVID States dataset does not include demographic information, only providing state-level estimates of mitigation behavior uptake with irregular time intervals between data points. However, it shares similarities with the Delphi US CTIS data, by providing estimates of individuals who wear a masks outside their homes, work outside their home, avoid contact with others, use public transportation, and spend time with others outside their home. Additionally, we used county-level population estimates from the U.S. Census by sex and age, which we aggregated to the state-level when needed.

We validated our estimates of behavior uptake with three datasets that were not used to inform our predictions. Sanchez *et al*.^22^ surveyed 4,654 individuals between August 2020 and December 2020 assessing mask wearing, social distancing, hand sanitizer use, and hand washing at the national level. Taube *et al*.^37^ estimated county-level mask wearing from September 2020 and May 2021 using binomial regression models combined with survey raking and debiasing. YouGov^23^ provides national-level survey estimates on mask wearing, going to work, and avoiding others in public. Each of these datasets serve as benchmarks for evaluating our behavior uptake estimates.

### Data Cleaning

We addressed several data issues before estimating mitigation behavior, including aligning datasets and imputing missing values. The COVID States dataset reports data at uneven time intervals, which do not align with intervals used by the Delphi US CTIS data. We resolve this by interpolating the COVID States data using a first-order polynomial, creating a uniform time grid with a daily spacing. The grid’s origin is placed on April 1, 2020, the earliest date recorded in both the COVID States and Delphi US CTIS datasets. We then aggregated the daily estimates into monthly values to match the COVID States and Delphi US CTIS time aggregation, thus ensuring consistency across datasets. Similarly, we adjusted the U.S. Census data by aggregating age groups so they matched the age groups used in the Delphi US CTIS data.

The state-level Delphi US CTIS data contains missing observations of mitigation behavior uptake due to non-response and data entry errors. However, since these missing values are required for future analysis, we imputed them using the MissForest iterative imputation method^29^. This approach begins with a simple imputation method (e.g., mean-imputation) while tracking which values were imputed. Next, a random forest is trained on the imputed data to refine the predictions for missing values. This process of training a random forest on successively imputed data is repeated until a convergence criterion is met, or a maximum number of iterations have passed. We implemented MissForest in Python with sklearn^38^ using IterativeImputer and RandomForestRegressor. Our implementation used 100 trees per random forest, that are trained a maximum of 10 times.

We explored two imputation approaches: imputing all of the mitigation behaviors simultaneously and imputing them one-at-a-time under various levels of missingness (see Tables 4, 5). When missingness was low–corresponding to Scenarios 1–3, where only a subset of data within a state was removed–we found both methods performed similarly. However, in some cases imputing behaviors one-at-a-time resulted in better performance. Only under extreme missingness (Scenario 4), where all data from a single state were removed while retaining data from the remaining states (see Validation of MissForest), did imputing using all of the behaviors simultaneously consistently outperform the one-at-a-time method. Since our dataset did not exhibit such high levels of missingness, we opted to impute each behavior one-at-a-time. During imputation we include information about the date, demographics, and the state that the estimates pertain to, to capture correlations between these factors and behavioral data. Additionally, we require an estimate of the sample size for each imputed group so that percentages can be converted into counts of individuals; for this, we let the sample size equal 100.

### Iterative Proportional Fitting

With the data cleaned, we shift to producing estimates of mitigation behavior uptake. Our goal is to produce county-level estimates of the percentage of individuals who engage in five key behaviors: wearing a mask, going to work, avoiding contact with others, using public transportation, and spending time with others while accounting for their age and sex. To achieve this, we employ the iterative proportional fitting (IPF) algorithm^33–35^ following a two-step process similar to the one presented by Beckman *et al*.^36^. We generate county-level estimates of behavior for each mitigation behavior across age groups and sex categories for each month between September 2020 and June 2022, resulting in a total of 968,044 predictions per behavior.

#### Step 1: State-Level Estimation

The first step of the IPF process is estimating behavior uptake at the state-level. This requires a seed table and corresponding marginals for each state. The seed table is created from the imputed state-level Delphi US CTIS data, providing an initial sample of behavior uptake by age group and sex. To prevent numerical instability, each entry of the seed table is increased by 1 × 10^−6^, to ensure no entry has a zero value. The marginals include:

1. State population by age group and sex (from aggregated U.S. Census data).
2. Behavior uptake by age group (from Delphi US CTIS and interpolated COVID States data).
3. Behavior uptake by sex (from Delphi US CTIS and interpolated COVID States data).

To construct behavior marginals for each demographic (e.g., age group or sex) we define an element of the marginal as:

1 $${ {\mathcal M} }_{i}=\frac{{N}_{i}}{{N}_{total}}\times {\alpha }_{i}$$

where *N*_*i*_ is the population in demographic group *i*, *N*_*total*_ is the total population (both obtained from the U.S. Census), and *α*_*i*_ is the estimated behavior uptake for that group. Here, *i* indexes demographic groups (e.g., male vs. female or sequential age groups). The notation *i* + 1 refers to the next group in the ordered set of demographic categories. To ensure consistency between state-level Delphi US CTIS data and the constructed marginal, we impose the constraint:

2 $${\alpha }_{i+1}-{\alpha }_{i}={\delta }_{i},$$

where *δ*_*i*_ is defined deterministically as the difference between behavior uptake in demographic groups measured in the state-level Delphi US CTIS. The final marginal values are determined by ensuring that the weighted sum of the demographic groups adds to the estimated fraction of the total population, *z*, measured in the COVID States data. To complete this process, we solve

3 $$\sum \,_{i}{\alpha }_{i}\frac{{N}_{i}}{{N}_{total}}=z,$$

for *α*_0_, …, *α*_*i*_. This process preserves the relationship between behavior and demographics during the up sampling process. Once the marginals and seed table are established, we use IPF to produce estimates of behavior uptake for each state accounting for age and sex that is used in the next step to inform our county-level estimates.

#### Step 2: County-Level Estimation

The second step extends the estimation process to the county-level. For a given state, this step requires:

- A seed table initialized with uniform values (set to one) for behavior uptake across counties, age groups, and sex.
- Marginals describing:
  1. County population by age group and sex (from the U.S. Census).
  2. County-level behavior uptake by age group (constructed using state-level Delphi US CTIS data).
  3. County-level behavior uptake by sex (similarly constructed).
  4. Behavior uptake by age group and sex (obtained from the state-level IPF step).

When generating the county-level estimates, we ensure that differences between counties are maintained in the same way that they are matched for demographics (i.e., by solving Equation (3) where *α*_*i*_ represents behavior uptake in county *i*, *N*_*i*_ and *N*_*total*_ represent total population for country *i* and the given state, respectively, both obtained from the U.S. Census, and *z* represents state level behavior uptake). To address missing county-level in the Delphi US CTIS data, we impute missing values using the 2020 U.S. Census county-level Urban and Rural classification. If data is unavailable for a county, we substitute the average behavior uptake for counties of the same classification within the state. If there is not enough data at that level, we default to the state-level estimate.

#### Handling Edge Cases

In both IPF steps, we default to using the state-level estimate when it’s not possible to preserve differences between counties or demographic groups. This situation arises, for example, when the observed difference between two groups is larger than the total estimate for the state. During the IPF process, we track convergence by recording the number of estimates with an error greater than 1 × 10^−6^ between the estimated and true marginals. For most behaviors, including mask wearing, going to work, avoiding contact with others, and spending time with others, all estimates achieved errors below the 1 × 10^−6^ threshold. Performance was weaker for public transportation use, particularly in counties with limited data availability. In total, IPF failed to converge in 14 instances for this behavior. This limitation likely reflects sparsity in reported behavior uptake; however, we retain these estimates for completeness. We report the corresponding state–date pairs in Table 2, along with the maximum error between estimated and target marginals. This maximum discrepancy represents the absolute difference in the number of individuals aggregated across all counties within a state. Of the 14 non-convergent cases, seven exhibit discrepancies exceeding one individual, with the largest discrepancy reaching 345 individuals at the state level.

**Table 2:** Estimates of public transportation use that failed to converge in IPF.

state_fip	date	max error (individuals)
2	20220201	7 × 10^−6^
5	20210501	138
5	20220201	194
10	20211201	2 × 10^−4^
16	20220301	1
28	20211001	345
30	20220201	1 × 10^−4^
33	20200901	2
35	20201001	141
35	20220601	1 × 10^−6^
46	20210301	23
56	20220101	6 × 10^−4^

In total, IPF failed to converge 14 times for the public transportation use. Of these failures, seven represent a discrepancy of at least 1 person. These errors are measured across all county-level estimates associated with these state-date pairs.

## Data Records

The data^39^ produced for this study are publicly available at Zenodo (https://zenodo.org/records/15177217). All data are stored in a single CSV file containing 11 columns and 968,044 rows. We show the structure of the dataset in Table 3, where the columns are defined as follows:

- “**date**”: The month for which behavior uptake estimates were generated, formatted as YYYYMMDD.
- “**state_fip**”: The U.S. Census state-level Federal Information Processing Standards (FIPS) code, serving as a geographic identifier.
- “**county_fip**”: The U.S. Census county-level FIPS geographic identifier.
- “**sex**”: The sex of the population subgroup described in the row (Female or Male).
- “**age**”: The age group described in the row (categorized as 18–24, 25–34, 35–44, 45–54, 55–64, 65–74, 75plus).
- “**N**”: The total number of individuals that belong to the described demographic subgroup.
- “**wear_mask**”: The estimated percentage of individuals (out of N) who report wearing a mask outside of their home.
- “**avoid_contact**”: The estimate percentage of individuals (out of N) who report avoiding contact with others outside their home.
- “**spend_time**”: The estimated percentage of individuals (out of N) who report spending time with others outside of their home.
- “**public_transit**”: The estimated percentage of individuals (out of N) who report using public transportation.
- “**work_outside_home**”: The estimate percentage of individuals (out of N) who report working outside of their home.

**Table 3:** Sample of behavior uptake from the dataset by county, age group, and sex over time.

date	state_ fip	county_ fip	sex	age	N	wear_ mask	avoid_ contact	spend_ time	public_ transit	work_ outside_ home
20200901	1	61	Male	18–24	948	0.602	0.362	0.618	0.006	0.559
20200901	1	61	Male	25–34	1505	0.591	0.308	0.597	0.010	0.629
20200901	1	61	Male	35–44	1519	0.548	0.381	0.605	0.010	0.580
20200901	1	61	Male	45–54	1746	0.541	0.363	0.594	0.006	0.566

Behavior uptake values are truncated to three decimal places for readability, but the full dataset remains full precision.

## Technical Validation

### Validation of MissForest

We test our imputation strategy by examining how well MissForest imputed synthetically introduced missing data in the state-level Delphi US CTIS dataset. This process requires a complete dataset; however, because no state had complete data for the “avoiding contact” behavior, we excluded this variable from our validation study. After removing the avoiding contact behavior, we are left with 21 states with complete data for the remaining behaviors. With this complete dataset, we examined four missingness scenarios:

1. Scenario 1: Removal of all data for males aged 18–24 in a single state.
2. Scenario 2: Removal of all data for females aged 75 plus in a single state.
3. Scenario 3: Removal of all data for all individuals aged 18–24 in a single state.
4. Scenario 4 (worst-case): Removal of all data for a single state.

The first three scenarios reflect similar missingness that we observed in our data, while the final scenario is a worst case scenario to test robustness. For each scenario, we introduced synthetic missing data for one state at a time and then imputed the missing values using the remaining 20 states.

#### Imputation Performance Metrics

To quantify imputation accuracy, we calculated the mean absolute error (MAE), root mean squared error (RMSE), and mean absolute percentage error (MAPE) defined as,

4 $$MAE=mean\,[abs({X}_{true}-{X}_{imp})],$$

5 $$RSME=\sqrt{mean\,[{({X}_{true}-{X}_{imp})}^{2}]},and$$

6 $$MAPE=mean\,[abs(\frac{{X}_{true}-{X}_{imp}}{{X}_{true}})],$$

where *X*_*imp*_ are the imputed values, *X*_*true*_ are their true counterparts, *abs* is the absolute value, and *mean* is the empirical mean. Note that MAE measures average absolute differences, RMSE penalizes larger deviations more heavily, and MAPE normalizes errors as a percentage.

We show the RMSE, MAE, MAPE for the imputed behaviors averaged over the 21 states in the complete dataset when using all behaviors and a single behavior for each missingness scenario in Tables 4, 5, respectively. The results show notable patterns in the imputation performance depending on the extent of missing data and whether multiple behavioral variables or a single variable were used for imputation.

**Table 4:** Imputation validation using all behaviors at once.

(a) Wearing mask			
Senario	RMSE	MAE	MAPE
one	7.004	5.512	0.134
two	5.497	4.014	0.071
three	6.961	5.353	0.120
four	7.915	5.952	0.119

(b) Going to work			
Scenario	RMSE	MAE	MAPE
one	5.003	3.975	0.069
two	1.077	0.858	0.146
three	4.569	3.617	0.065
four	3.040	2.298	0.076

(c) Using public transportation			
Scenario	RMSE	MAE	MAPE
one	3.627	2.81	0.282
two	0.690	0.578	0.413
three	3.151	2.432	0.290
four	2.906	2.307	0.480

(d) Spent time with others outside of household.			
Scenario	RMSE	MAE	MAPE
one	4.589	3.671	0.066
two	2.516	2.058	0.058
three	4.027	3.172	0.058
four	2.941	2.320	0.054

We explore the ability of MissForest to impute synthetically introduced missing data in four scenarios applied to 21 states. Each scenario is applied to a single state at a time and include: (one) removing data for males between 18 and 24 years old, (two) removing females that are 75 plus years old, (three) anyone between 18 and 24 years old, (four) removing all data. The average root mean squared error (RMSE), mean average error (MAE), and mean average percentage error (MAPE) between the imputed and true values are reported.

**Table 5:** Imputation validation using behaviors one at a time.

(a) Wearing mask			
Scenario	RMSE	MAE	MAPE
one	4.775	3.859	0.097
two	2.622	1.920	0.033
three	4.672	3.602	0.084
four	11.480	9.288	0.193

(b) Going to work			
Scenario	RMSE	MAE	MAPE
one	6.996	5.983	0.101
two	1.219	0.947	0.163
three	6.570	5.531	0.099
four	4.772	3.702	0.110

(c) Using public transportation			
Scenario	RMSE	MAE	MAPE
one	3.239	2.513	0.253
two	0.943	0.808	0.620
three	3.270	2.602	0.318
four	3.175	2.519	0.526

(d) Spent time with others outside of household.			
Scenario	RMSE	MAE	MAPE
one	5.926	4.928	0.090
two	2.285	1.870	0.052
three	5.772	4.837	0.090
four	4.833	4.042	0.093

We explore the ability of MissForest to impute synthetically introduced missing data in four scenarios applied to 21 states. Each scenario is applied to a single state at a time and include: (one) removing data for males between 18 and 24 years old, (two) removing females that are 75 plus years old, (three) anyone between 18 and 24 years old, (four) removing all data. The average root mean squared error (RMSE), mean average error (MAE), and mean average percentage error (MAPE) between the imputed and true values are reported.

When comparing the errors in scenarios with lower amounts of missing data (Scenarios 1–3), we observe that using a single variable imputation often performs as well as or outperforms using all of the behaviors. In these cases, MissForest was able to recover the missing values with high accuracy, and the average imputation error remained relatively low. However, note that we did not observe such large amounts of missingness in our data. Across these scenarios, the RMSE and MAE are consistent, suggesting there are no large outliers in our predictions. The imputed values were, on average, within approximately six percentage points of the true values. For example, if the true value was 50%, the imputed estimates typically ranged between 44% to 56%, showing a reasonable level of precision. While the RMSE and MAE values remained relatively stable, the MAPE values varied more significantly, particularly for behaviors such as public transit use, where the overall proportion of in dividuals engaging in this activity was low.

In the worst-case scenario (Scenario 4), where all data for a single state were removed, imputation performance declined for some behaviors and evaluation metrics, although the extent of degradation varied across behaviors. For instance, mask wearing exhibited larger increases in RMSE under single-variable imputation, whereas lower-prevalence behaviors such as public transportation showed smaller changes in absolute error but greater variability in MAPE. These differences likely reflect variation in baseline prevalence and temporal stability across behaviors, as well as the sensitivity of MAPE to small denominators. Given that real-world missingness in our dataset aligns more closely with Scenarios 1–3, our findings suggest that MissForest provides reliable and largely unbiased imputations under typical conditions. While the worst-case scenario highlights potential performance degradation for certain behaviors and metrics, such extreme missingness is not present in our dataset. As a result, we expect the imputation procedure to yield realistic and robust estimates for the analyses presented here.

### Validation of IPF

We validated our IPF-generated estimates using multiple approaches to ensure both accuracy and reliability. First, we confirmed that all imputed estimates were non-negative and that our fitted marginals matched the target ones. For all behavioral variables except using public transportation, we found that our fitted marginals agreed with the expected ones within an absolute error of 1 × 10^−6^. These checks ensure that the results we have generated are consistent with reality.

**Fig. 2:**
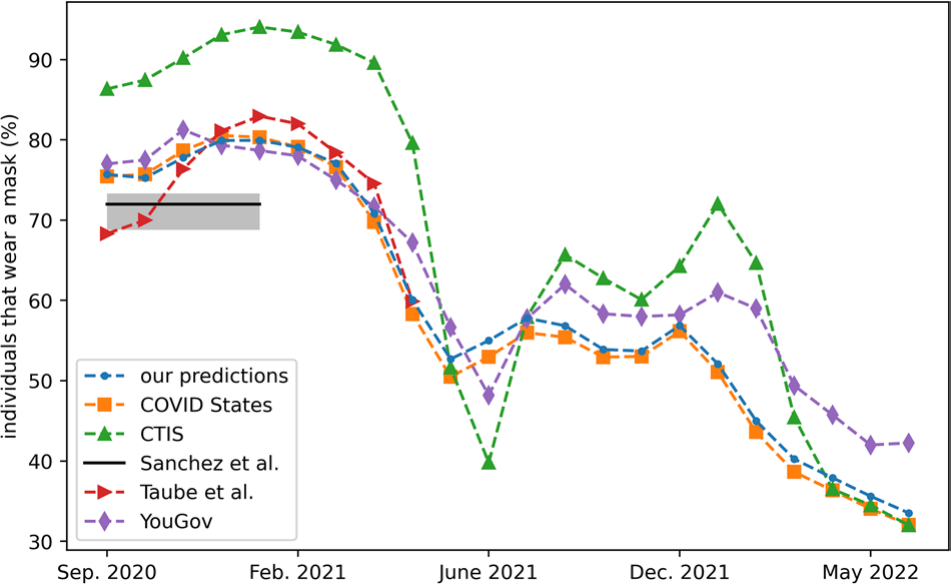
Comparison of national estimates of mask wearing behavior between September 2020 and June 2022. Our estimates capture the overall trend of national mask wearing behavior measured by various surveys.

To further assess the performance of IPF, we aggregated the state-level estimates and compared them against national-level behavioral data from both training datasets (Delphi US CTIS and COVID States) as well as external sources, including^22,23,37^. In Fig. 2, we show a comparison of national mask-wearing behavior between September 2020 and June 2022. Our model estimates are shown in blue, alongside national trends from Delphi US CTIS (green), COVID States (orange), YouGov (purple), and Taube *et al*. (red). Additionally, we include a 95% confidence interval from Sanchez *et al*., shown in black. Note that estimates generated by Sanchez *et al*.^22^ and Taube *et al*.^37^ do not span the entire time window, thus, they appear only for partial periods. Additionally, Sanchez *et al*. reports a single cross-sectional estimate collected between August and December 2020 and does not provide finer temporal resolution. Accordingly, we display it as a constant value over the overlapping period; although visually distinct, its magnitude is consistent with the other datasets.

Our national estimates of mask-wearing behavior fall in the center of all existing predictions, and are consistent with the overall trend observed across all datasets. Notably, our estimates most closely match those from COVID States, which is expected as our IPF procedure was constrained by that dataset. Although our estimates are slightly higher than those reported from Sanchez *et al*., this pattern is also evident in other datasets. However, when comparing to the estimates from Taube *et al*., we see our results are largely consistent, further validating our approach.

**Fig. 3:**
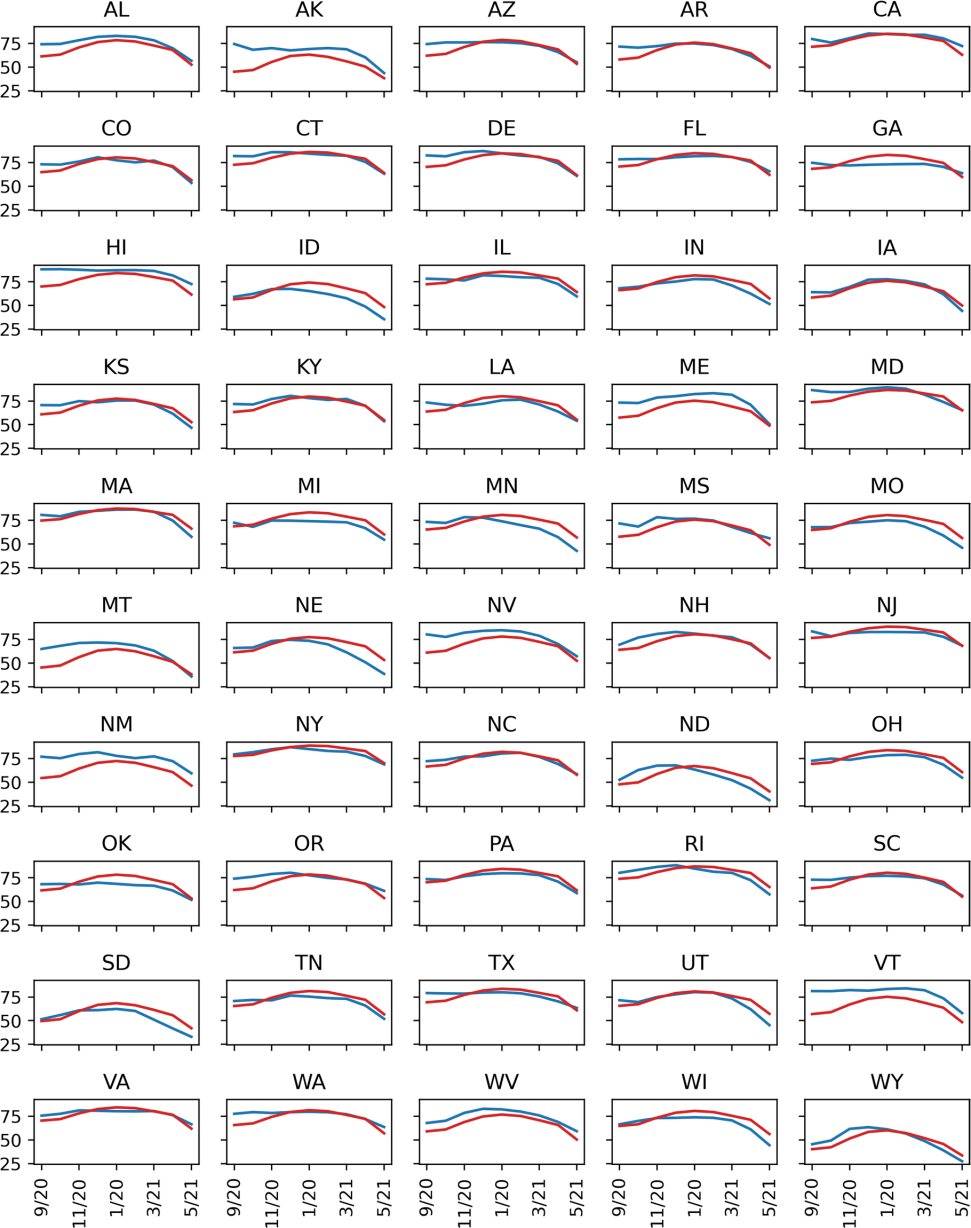
State-by-state comparison of estimates of statewide mask wearing behavior from this work (**blue**) and Taube *et al*. (**red**) between September 2020 and May 2021. For the majority of states, our estimates and those from Taube *et al*. are consistent.

We extended the validation by performing state-level comparisons with the data from Taube *et al*. In Fig. 3, we show state-level estimates of our predictions (blue) and those from Taube *et al*. (red) of mask wearing behavior from September 2020 to June 2022. Across all states, the general trends in behavior are consistent between the two datasets, and in most cases, the predicted values are quantitatively similar, reinforcing the model’s ability to capture geographic variability.

**Fig. 4:**
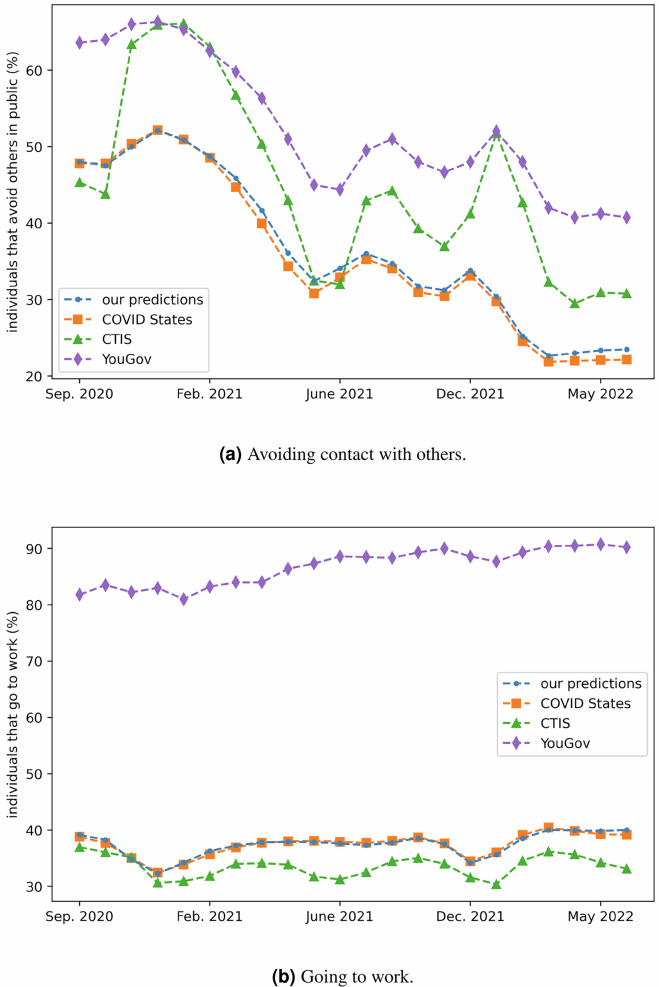
Comparison of (**a**) avoiding contact with others and (**b**) going to work at a national level. Our estimates are consistently lower than those from YouGov, though they capture the overall trend. This is because our predictions are constrained by the COVID States datasets, which also show lower values than YouGov. The true population values therefore remain uncertain and may lie somewhere between our estimates and YouGov’s reported values.

To evaluate other behaviors, we compared our national-level estimates for “avoiding contact with others” and “going to work” against those from YouGov. Figure 4 displays these comparisons, showing our estimates (blue), YouGov (purple), COVID States (orange), and Delphi US CTIS (green). While our model systematically underpredicts the values reported by YouGov, the temporal trends remain consistent. Interestingly, the Delphi US CTIS and COVID States estimates, which are derived from independent data sources, also fall below the YouGov estimates. This suggests that the discrepancy may result from systematic overestimation in the YouGov data rather than underestimation by our model.

Overall, the results of our validation studies show that our mask-wearing estimates are consistent with external benchmarks, both at the national and state levels. For other behaviors, while point estimates diverge somewhat from YouGov, the consistency in trends and the agreement between COVID States and Delphi US CTIS support the reliability of our imputed values. Together, these findings suggest that the IPF-based estimates are both reasonable and robust. Moreover, the alignment of our model with our national and state-level data gives us confidence that our finer-scale estimates, by county and demographic group, are also reliable for downstream analyses.

While our validation exercises demonstrate consistency with multiple independent data sources, we emphasize that the released dataset does not include formal uncertainty intervals. The available survey summaries do not provide sufficient information to recover the full covariance structure necessary for principled uncertainty propagation. Therefore, downstream users should interpret these estimates as behavior reconstructions constrained to match published marginals, rather than as probabilistic draws from a fully specified statistical model.

## Usage Notes

The monthly temporal resolution of this dataset is intended to capture medium-term behavioral trends rather than short-term fluctuations. As such, these estimates are most appropriate for:

1. informing lower-frequency behavioral regimes in transmission models,
2. parameterizing behaviorally stratified simulations where monthly updates are sufficient,
3. supporting retrospective analyses of spatial and demographic heterogeneity in behavior, and
4. serving as calibration targets for models incorporating endogenous behavior.

These data are not intended to capture rapid behavioral changes occurring at weekly or daily time scales, such as those following immediate policy shifts.

Although this study focuses on COVID-19, the underlying methodological framework is generalizable to other infectious diseases. Applying this approach to a different pathogen would require: (1) survey or observational data capturing behaviors relevant to that disease’s transmission pathway (e.g., contact patterns, hygiene practices, vaccination uptake), (2) demographic information aligned with available population marginals (e.g., age, sex), and (3) consistent spatial aggregation between behavioral data and census data. The temporal resolution of the resulting estimates would depend on the frequency and stability of available input data; higher-frequency behavioral data could be incorporated directly within the same framework. Thus, the approach provides a scalable mechanism for integrating heterogeneous behavioral datasets into spatially and demographically structured infectious disease simulations.

Because full survey covariance information is not available, the dataset provides point estimates conditioned on published survey marginals and demographic population counts. These estimates inherit upstream sampling variability from the source surveys; however, formal confidence intervals are not provided. As a result, the estimates are most appropriate for scenario exploration, behavioral parameterization, and retrospective analysis rather than formal statistical inference requiring explicit uncertainty quantification. Finally, we assume independence across behaviors: for example, the probability that an individual wears a mask is treated as independent of the probability that the same individual avoids others in public settings.

## Data Availability

Our data^39^ is available from Zenodo (https://zenodo.org/records/15177217) under a Creative Commons Attribution 4.0 International license as a single CSV file.

## Code availability

The code used to generate our estimates is available from GitHub (https://github.com/lanl/epicast_heterogeneous_behavior). All of our analyses were performed with Python 3.11.7.

## References

[1] Nikolaou, M. Revisiting the standard for modeling the spread of infectious diseases. *Scientific Reports* **12**, 7077 (2022). 35490159 10.1038/s41598-022-10185-0 PMC9056532

[2] Casagrandi, R., Bolzoni, L., Levin, S. A. & Andreasen, V. The sirc model and influenza a. *Mathematical biosciences* **200**, 152–169 (2006). 16504214 10.1016/j.mbs.2005.12.029

[3] Hiram Guzzi, P., Petrizzelli, F. & Mazza, T. Disease spreading modeling and analysis: A survey. *Briefings in Bioinformatics* **23**, bbac230 (2022). 35692095 10.1093/bib/bbac230

[4] Ross, R. Some quantitative studies in epidemiology. *Nature* **87**, 466–467 (1911).

[5] Anderson, R. M. & May, R. M.*Infectious diseases of humans: dynamics and control* (Oxford university press, 1991).

[6] Hethcote, H. W. The mathematics of infectious diseases. *SIAM review* **42**, 599–653 (2000).

[7] Germann, T. C., Kadau, K., Longini Jr, I. M. & Macken, C. A. Mitigation strategies for pandemic influenza in the united states. *Proceedings of the National Academy of Sciences* **103**, 5935–5940 (2006). 10.1073/pnas.0601266103 PMC1458676 16585506

[8] Venkatramanan, S. *et al*. Using data-driven agent-based models for forecasting emerging infectious diseases. *Epidemics* **22**, 43–49 (2018). 28256420 10.1016/j.epidem.2017.02.010 PMC5568513

[9] Tracy, M., Cerdá, M. & Keyes, K. M. Agent-based modeling in public health: current applications and future directions. *Annual review of public health* **39**, 77–94 (2018). 29328870 10.1146/annurev-publhealth-040617-014317 PMC5937544

[10] Bedson, J. *et al*. A review and agenda for integrated disease models including social and behavioural factors. *Nature human behaviour* **5**, 834–846 (2021). 34183799 10.1038/s41562-021-01136-2

[11] Funk, S., Salathé, M. & Jansen, V. A. Modelling the influence of human behaviour on the spread of infectious diseases: a review. *Journal of the Royal Society Interface* **7**, 1247–1256 (2010). 20504800 10.1098/rsif.2010.0142 PMC2894894

[12] Ryan, M., Brindal, E., Roberts, M. & Hickson, R. I. A behaviour and disease transmission model: incorporating the health belief model for human behaviour into a simple transmission model. *Journal of the Royal Society Interface* **21**, 20240038 (2024). 38835247 10.1098/rsif.2024.0038 PMC11338573

[13] Verelst, F., Willem, L. & Beutels, P. Behavioural change models for infectious disease transmission: a systematic review (2010–2015). *Journal of The Royal Society Interface* **13**, 20160820 (2016). 28003528 10.1098/rsif.2016.0820 PMC5221530

[14] Weston, D., Hauck, K. & Amlôt, R. Infection prevention behaviour and infectious disease modelling: a review of the literature and recommendations for the future. *BMC public health* **18**, 1–16 (2018). 10.1186/s12889-018-5223-1 PMC5845221 29523125

[15] Morin, B. R., Fenichel, E. P. & CASTILLO-CHAVEZ, C. Sir dynamics with economically driven contact rates. *Natural resource modeling* **26**, 505–525 (2013). 25152563 10.1111/nrm.12011 PMC4139939

[16] Fu, F., Christakis, N. A. & Fowler, J. H. Dueling biological and social contagions. *Scientific reports* **7**, 43634 (2017). 28252663 10.1038/srep43634 PMC5333634

[17] Epstein, J. M., Parker, J., Cummings, D. & Hammond, R. A. Coupled contagion dynamics of fear and disease: mathematical and computational explorations. *PloS one* **3**, e3955 (2008). 19079607 10.1371/journal.pone.0003955 PMC2596968

[18] Valle, S. Y. D., Mniszewski, S. M. & Hyman, J. M. Modeling the impact of behavior changes on the spread of pandemic influenza. *Modeling the interplay between human behavior and the spread of infectious diseases* 59–77 (2013).

[19] Hu, T. *et al*. Human mobility data in the covid-19 pandemic: characteristics, applications, and challenges. *International Journal of Digital Earth* **14**, 1126–1147 (2021).

[20] Salomon, J. A. *et al*. The us covid-19 trends and impact survey: Continuous real-time measurement of covid-19 symptoms, risks, protective behaviors, testing, and vaccination. *Proceedings of the National Academy of Sciences* **118**, e2111454118 (2021). 10.1073/pnas.2111454118 PMC8713763 34903656

[21] The COVID States Project. https://lazerlab.shinyapps.io/Behaviors_During_COVID/.

[22] Sanchez, T. *et al*. Prevalence of covid-19 mitigation behaviors in us adults (august-december 2020): nationwide household probability survey. *JMIR Public Health and Surveillance* **9**, e37102 (2023). 38055314 10.2196/37102 PMC10702689

[23] YouGov. Personal measures taken to avoid COVID-19. https://yougov.com/en-us/articles/29430-personal-measures-taken-avoid-covid-19. Accessed: August 20, 2024.

[24] MacIntyre, C. R. *et al*. Mask use, risk-mitigation behaviours and pandemic fatigue during the covid-19 pandemic in five cities in australia, the uk and usa: A cross-sectional survey. *International Journal of Infectious Diseases* **106**, 199–207 (2021). 33771668 10.1016/j.ijid.2021.03.056 PMC7985682

[25] Mansuri, F. M., Zalat, M. M., Khan, A. A., Alsaedi, E. Q. & Ibrahim, H. M. Estimating the public response to mitigation measures and self-perceived behaviours towards the covid-19 pandemic. *Journal of Taibah University Medical Sciences* **15**, 278–283 (2020). 32837504 10.1016/j.jtumed.2020.06.003 PMC7334963

[26] Probst, J. C., Crouch, E. L. & Eberth, J. M. Covid-19 risk mitigation behaviors among rural and urban community-dwelling older adults in summer, 2020. *The Journal of Rural Health* **37**, 473–478 (2021). 34096648 10.1111/jrh.12600 PMC8242629

[27] Kassas, B., Morgan, S. N., Lai, J. H., Kropp, J. D. & Gao, Z. Perception versus preference: The role of self-assessed risk measures on individual mitigation behaviors during the covid-19 pandemic. *Plos one* **16**, e0254756 (2021). 34347778 10.1371/journal.pone.0254756 PMC8336792

[28] Bradley, V. C. *et al*. Unrepresentative big surveys significantly overestimated us vaccine uptake. *Nature* **600**, 695–700 (2021). 34880504 10.1038/s41586-021-04198-4 PMC8653636

[29] Stekhoven, D. J. & Bühlmann, P. Missforest–non-parametric missing value imputation for mixed-type data. *Bioinformatics* **28**, 112–118 (2012). 22039212 10.1093/bioinformatics/btr597

[30] United States Census Bureau. cc-est2021-agesex-all.csv (2023).

[31] United States Census Bureau. cc-est2023-agesex-all.csv (2023).

[32] United States Census Bureau. 2020_UA_COUTNY.xlsx (2020).

[33] Naszodi, A. The iterative proportional fitting algorithm and the nm-method: solutions for two different sets of problems. *arXiv preprint arXiv:2303.05515* (2023).

[34] Lovelace, R., Birkin, M., Ballas, D. & Van Leeuwen, E. Evaluating the performance of iterative proportional fitting for spatial microsimulation: new tests for an established technique. *Journal of Artificial Societies and Social Simulation* **18** (2015).

[35] Choupani, A.-A. & Mamdoohi, A. R. Population synthesis using iterative proportional fitting (ipf): A review and future research. *Transportation Research Procedia* **17**, 223–233 (2016).

[36] Beckman, R. J., Baggerly, K. A. & McKay, M. D. Creating synthetic baseline populations. *Transportation Research Part A: Policy and Practice* **30**, 415–429 (1996).

[37] Taube, J. C., Susswein, Z. & Bansal, S. Spatiotemporal trends in self-reported mask-wearing behavior in the united states: Analysis of a large cross-sectional survey. *JMIR Public Health and Surveillance* **9**, e42128 (2023). 36877548 10.2196/42128 PMC10028521

[38] Pedregosa, F. *et al*. Scikit-learn: Machine learning in Python. *Journal of Machine Learning Research* **12**, 2825–2830 (2011).

[39] Butts, D. J., Parikh, N. K. & Del Valle, S. Y. Data for “Heterogeneous estimations of non-pharmaceutical mitigation behavior during the COVID-19 pandemic” [Data set]. *Zenodo*10.5281/zenodo.15177217 (2025). 10.1038/s41597-026-07348-3 PMC13341756 42069745

